# Introduction: Human Animal Health in Medical Anthropology

**DOI:** 10.1111/maq.12488

**Published:** 2019-02-27

**Authors:** Hannah Brown, Alex M. Nading

**Affiliations:** ^1^ Department of Anthropology Durham University; ^2^ Watson Institute for International and Public Affairs Brown University

**Keywords:** humans, animals, medical anthropology, health

## Abstract

This introductory article maps out the parameters of an emerging field of medical anthropology, human animal health, and its potential for reorienting the discipline. Ethnographic explorations of how animals are implicated in health, well‐being, and pathogenicity allow us to revisit theorizations of central topics in medical anthropology, notably ecology, biopolitics, and care. Meanwhile, the conditions of the Anthropocene force us to develop new tools to think about human animal entanglement. Anthropogenic change reorients debates around health and disease, but it also requires us to move beyond what some consider the traditional boundaries of the discipline. Zoonotic diseases, veterinary medicine, animal therapeutics, and food and farming are examples of topics that force such movement.

Nonhuman animals are our partners in everyday life. As pets, livestock, and wildlife, animals are thoroughly embedded in practices of cultivation, consumption, and co‐habitation. This has important implications for health and healing. The safety and abundance of our food are dependent on the welfare of livestock, yet concerns about the rise of antimicrobial resistance reveal potentially catastrophic intersections between intensive animal production and new forms of pathogenicity. Contact with wild and domestic animals can create opportunities for pandemic outbreaks. Human disturbances to animal habitats are blamed for an ongoing mass extinction of animals. There is growing evidence of the contribution that companion and therapy animals make to emotional and physical well‐being. Living well with other animals is essential for the welfare of all species. Indeed, it is likely among the most pressing and urgent issues of our time.

This collection outlines an emerging area of medical anthropology: human animal health. The contributors explore issues of human–animal contact, co‐habitation, and separation in contexts where established intellectual paradigms are challenged by new ecologies, actors, and problems. By revealing both how biopolitics extends beyond the human and how practices of care cross species boundaries, human animal health reorients our understanding of well‐worn concerns in medical anthropology. At the same time, a view of health as more than human productively disturbs existing disciplinary settlements. The contributors build on a small but growing literature that has made the human interface central in questions of public health, clinical medicine, nutrition, and laboratory science. Still, many of the contributors found themselves surprised to be writing material that could be classed as medical anthropology. Thinking about the fecal remainders of animal production (Blanchette), the ecologies of hookworms (Lorimer), or the euthanasia practices of veterinary doctors (Hurn and Badman‐King) drew them into conversations within a subdiscipline in which they were not entirely comfortable. In assembling this collection, we consciously invited unfamiliar voices and perspectives as a way of exploring how thinking with nonhuman animals allows us to look anew at concepts of health, well‐being, and pathogenicity, as well as to question our own sense of intra‐disciplinary coherence.

This collection, then, aims to diversify medical anthropology by putting it into dialogue with other subdisciplines, but we also see human animal health as an opportunity for medical anthropologists to contribute to emerging debates in other areas, particularly as regards the recent turn to “multispecies ethnography.” While anthropological accounts of human–animal relations often celebrate multi‐species intimacy and sociality (see Davidson et al. 2005; Haraway 2008; Kirksey and Helmreich 2010; Tsing 2015), this collection complicates such narratives. Contact with animals certainly can be beneficial for human and animal well‐being, but it can also be dangerous. Public health interventions frequently emphasize reducing interactions between humans and animals. As a result, epidemiologists, policymakers, and researchers seeking to improve health outcomes often fail to recognize the depths, intensities, and affective complexities of social relations between humans and animals. This collection offers viewpoints that are neither overly determined by epidemiological narratives of disease spread, with a focus on the technological mitigation of pathogenic “spillover,” nor excessively saturated by a sentimentality of mutuality and entanglement. Instead, contributors attend to the ethnographic particularities of contexts where multispecies well‐being is enabled (and sometimes harmed) across intimate, institutional, and governmental scales.

In this introduction, we sketch the parameters of human animal health. We begin by outlining in more detail how thinking about humans and animals might disturb medical anthropology's existing boundaries. Such disturbance can provide new leverage on three key concepts for the subdiscipline: ecology, biopolitics, and care. We go on to highlight the different ways in which the empirical work of the authors in this collection enriches our understanding of these concepts.

## Reframing Medical Anthropology

Ecological thinking has a long history in medical anthropology, anchoring both classic introductory texts and recent work (e.g., McElroy and Townsend 2014 [original edition 1977]; Singer 2014). Ecological approaches emerged during the latter half of the 20th century, just as medical anthropologists were beginning to turn attention from a narrow focus on ethnomedicine to the political economy of health (Baer et al. 1997; Turshen 1984). This latter approach often attended explicitly to how land degradation, economic policies, and planning initiatives led to human suffering. For example, Farmer's (1992) pioneering study of AIDS in Haiti was partly a work of political ecology, tracing the epidemic to a catastrophic landscape change engendered by a dam project. Elsewhere, studies of malaria (Manderson 1992; Packard and Brown 1997) and dengue (Whiteford 1997; Winch et al. 1991) illustrated links between international health interventions such as DDT programs, the uneven provision of housing and infrastructure through state‐level welfare programs, and intimate encounters between humans and mosquitoes. These and other examples show how human attempts to deploy ecological knowledge instrumentally—whether to produce hydroelectric energy, to disrupt the lifecycles of insects with chemicals, or to produce livestock and poultry in economies of scale—tend to result in unintended social and environmental catastrophe as often as they result in balance or control (Ali and Keil 2011; Lowe 2010; Nading 2017). Even if interspecies relations were not directly theorized, in retrospect, critical medical anthropology is replete with reminders that human–animal relations are deeply implicated in many aspects of health and well‐being.

Today, there is a growing consensus that catastrophe is becoming a global norm. Human manipulation of Earth's ecosystems has engendered a new geological era, known by the now‐familiar name “Anthropocene” (Crutzen and Stoermer 2000). The conditions of the Anthropocene mean that the attentions of different scientific disciplines are beginning to converge around shared problems in attempts to understand interactions between humans and environments. Medical anthropologists are, of course, no strangers to disciplinary convergence. In fact, disease ecology has historically been a fruitful meeting point for ethnographers, epidemiologists, and policymakers, particularly in the control of zoonotic and vector‐borne diseases. In the Anthropocene, however, disciplinary convergence has become not just a technical issue but a thoroughly moral problem, laced with questions of responsibility, as well as “response‐ability” (Latour 2014, 138, citing Haraway). The question has now become not just “how should we act?” but “who can act, and how?” The temporal urgency of climate change has reignited the longstanding question of the political and moral purpose of ethnography in public health (e.g., Adams et al. 2014; Janes and Corbett 2009; Kleinman 2010; Pigg 2013), particularly since so many anthropologists do their work in contexts where possibilities for human flourishing are deeply constrained not only by rising temperatures and sea levels but also by deep histories of economic inequality, colonialism, racism, and other conditions that are not of people's choosing (Farmer 2004; Kleinman et al. 1997; Singer et al. 1992). In the contemporary period, economics and ecology converge to constrain and enable human agency in new ways.

The broad recognition of this critical moment in (human) planetary time finds medical anthropologists laboring on new terrain. Anthropologists have begun to question the completeness implied by ecological portrayals of human and nonhuman behavior that are reliant on the heuristic of the “system”—an abstract rendering of relations between human beings, animals, plants, and microbes. In systems thinking, humans and other species act on one another as discrete, complete entities. Systems thinking has proved unsatisfying to anthropologists who wish to respond to the call to consider the moral responsibilities and “response‐abilities” species have to one another. Seeking an alternative, anthropologists have begun to explore how humans and other species are thoroughly entangled with one another. From the new science of the microbiome to studies of vector‐borne disease, interspecies *relationships*, rather than individual species, have become key units of analysis (Helmreich 2009; Livingston and Puar 2011; Nading 2014; Porter 2013). Such an approach raises ontological questions about life itself: about what makes humans human, animals animal, and microbes microbial. If all living creatures are to some extent symbionts, then they can never be satisfyingly approached as constituents of abstract systems.

In the Anthropocene, these ontological questions blend with issues of political economy, sometimes in surprising ways. Contributors to this volume highlight that multispecies health is characterized by new clashes and convergences between ecology and economics, for example within industrialized farming (Blanchette, Keck), and even within our own bodies (Lorimer). The ecological approach pioneered by an earlier generation of medical anthropologists was of course keenly attuned to the political economic dimensions of interspecies encounter. Given its continued commitment to addressing the effects of inequality, then, our subdiscipline is particularly well positioned to suture the ontological questions that attend the recent turn to multispecies response‐ability with the abiding political economic questions that spurred the explosion of critical medical anthropology over the last quarter of the 20th century. In human animal health, we cannot separate analyses of the violence of the political economic structures in which we live from debates about who we are, and what is knowable. We must interrogate questions that are simultaneously ethical, ontological, and economic.

To summarize, our first major point is that—as with other approaches concerned with understanding the future of life in the Anthropocene—human animal health reanimates questions of responsibility, while simultaneously disturbing boundaries between ontological and political economic dimensions of life. In human animal health, old distinctions between Marxist and interpretivist anthropology, and indeed between critical and applied approaches, are collapsed.

This leads to our second point: Thinking about human animal health involves developing a more than human conceptualization of biopolitics. There is, of course, a long history of nonhuman animals figuring prominently in regimes of governmental attention and intervention. Malaria control is a good example. Malaria interventions have targeted domestic organization (Panter‐Brick et al. 2006) and clinical practice (e.g., Chandler et al. 2008), involved large‐scale environmental changes to eradicate breeding spaces (Packard 2007), included the coordinated use of pesticides such as DDT, and encompassed city planning initiatives that have entangled disease control within broader attempts to manage race, work, gender, and citizenship (Curtin 1985; Kelly and Lezaun 2014).

Within contemporary global health, however, new kinds of multispecies biopolitics are emerging. The most prominent recent governmental response to the question of how to achieve well‐being responsibly within entangled, multispecies environments has been the rise of the “One Health” agenda. One Health interventions seek to harness “the collaborative efforts of multiple disciplines … to attain optimal health for people, animals, and our environment” (AMVA 2008). While “one medicine” approaches have a long history, One Health is a more recent endeavor, spurred by what its proponents see as increased and intensified interactions between humans, animals, and environments—what we might describe as the conditions of the Anthropocene (Zinsstag et al. 2011).

This is not a simple question of extending biopolitical attention to new domains. One Health is marked by forms of contemporary governmentality that erase distinctions between state, non‐state, public, and private institutions and create new opportunities for profit seeking within health (Lachenal 2014). In telling fashion, participants at the 2004 conference convened by the Wildlife Conservation Society that resulted in a joint call to action around human–animal health, sought to trademark the term One World One Health^TM^ (Woldehanna and Zimicki 2015, 88; Zinsstag et al. 2011). Funding programs for One Health are often substantial. For example, the PREDICT consortium based at UC Davis, which seeks to identify emerging pathogens in animal hosts and strengthen response capacity to future outbreaks, has received over $100 million in funding since 2009. We are drawn to make comparisons with health expenditure in countries where we have recently carried out fieldwork; in 2014, annual public and private health expenditure combined was $224 per capita in Sierra Leone and $445 per capita in Nicaragua (see https://en.wikipedia.org/wiki/List_of_countries_by_total_health_expenditure_per_capita). As in other global health contexts, then, One Health programs wield a degree of power and influence that changes the contours of health governance (Chien 2013). New kinds of “global assemblages” (Ong and Collier 2006) involving state and non‐state partnerships are emerging, characterized by shifting forms of “sovereign responsibility” in which a range of actors claim the right to manage interventions, monitor spending, and determine beneficiaries (Brown 2015).

One Health thereby acts as what Star and Griesemer (1989, 387) call a “boundary object”: simultaneously “adaptable to different viewpoints and robust enough to maintain identity across them.” Indeed, many anthropologists have cautiously embraced One Health and push for greater social science involvement in One Health initiatives, seeing possibilities to attend to new dimensions of inequalities revealed at human–animal interfaces within enlarged understandings of pathology (Dzingirai et al. 2017; Rock et al. 2009). Others have identified opportunities for “critical and constructive social science engagement with One Health” (Craddock and Hinchliffe 2015, 1).

Critical engagement with One Health suggests that projects often falter in translating across international, national, and local scales (e.g., Smith et al. 2015), partly because they involve organizing logics (“One‐isms”) that undervalue diversity and locally contingent practices involved in the production of health (Hinchliffe 2015; MacGregor and Waldman 2017). Such problems are understandable, given that funding structures for One Health have tended to favor the kinds of abstract ecosystem modeling discussed above. The undervaluing of the diverse ways animals are implicated in human health means that One Health projects have generally focused on animals as carriers of diseases that might cause harm to humans rather than as companions, coworkers, or even kin (Rock 2017). One Health initiatives have also tended to favor a predictive approach to disease transmission over a close analysis of the affective and symbolic (not to mention political and economic) dimensions of human–nonhuman relations. For example, farmers often reject biosecurity measures that are aimed at making animals healthier but entail misunderstandings of what matters to farmers in terms of how animals should be raised and for what purposes (Keck 2008; Porter 2013).

A final point at which human animal health provides new leverage in medical anthropology is through the concept of care. How to define care, how to study it ethnographically, and how it varies culturally are questions that have animated medical anthropology in recent years (e.g., Latimer 2013; Mol 2008; Taylor 2008). Care's relational, pragmatic dimensions present an alternative calculus to classical ethics, based upon utilitarian principles of well‐being (Gilligan 1993 [1982]; Mol et al. 2010). Care has been intensively theorized in contexts from food and nutritional practices (Paxson 2008;Yates‐Doerr 2012) to clinical encounters (e.g., Brown 2012; Kleinman and van der Geest 2009; Read 2007). Yet care is undeniably also a transspecies endeavor (Puig de la Bellacasa 2017). Thinking about caring interactions with animals opens up questions not only of attachment but also of violence (Law 2010; van Dooren 2016), morality and religon frameworks (see Hurn and Badman‐King, this special issue), and forces us to confront the shared embeddedness of humans and animals within same highly unequal global political economy (see Parreñas 2012).

For example, when primates or rodents are animal models in laboratory experiments designed to develop pharmaceutical drugs, care for these animals becomes a mechanism for turning vital life processes of metabolism, immunity, and reproduction into what Haraway (2008, 45–68) calls “lively capital.” Mammals are not the only animals whose lives are being turned into capital. Disease‐carrying mosquitoes from the *Aedes* and *Anopheles* genuses, once vilified as enemies to human beings, are now being turned into tools for disease control. Transgenic manipulations have produced populations of mosquitoes that either cannot transmit diseases like dengue and malaria or cannot reproduce in the wild (Beisel and Boëte 2013; Lezaun and Porter 2015; Nading 2015). For these novel populations to be effective tools (and viable sources of lively capital), technicians must feed them and provide them with shelter. As Carrie Friese and Joanna Latimer discuss in this special issue, interspecies caring relationships in such experimental contexts are affectively charged, but they are never innocent.

Multispecies care is, of course, bidirectional. Animals can also care for humans. A failure to recognize the two‐way nature of interspecies care could constitute a form of what Tronto (1993) terms “privileged irresponsibility,” perpetuating political fantasies of autonomous, independent, and self‐reliant (human) individuals. Contributions to this special issue build on such insights in their consideration of the role of microbiota, pets, and livestock in regulating human health. Such animals care for humans by becoming valued members of extended kin networks (Porter and Hurn and Badman‐King, this special issue), by regulating immune response (Lorimer, this special issue), and by ordering agricultural systems. While these and other species provide for the well‐being of humans, their lives can also, under different circumstances, be reduced to a market exchange value. Thus, interspecies care is not the opposite of animal commodification, but a mutable position on a relational continuum (see Blanchette, this special issue).

Having outlined some of the ways in which human animal health raises new analytical questions in medical anthropology, we now turn our attention to four significant sites of enquiry. These are (1) zoonotic diseases; (2) veterinary anthropology; (3) animal therapeutics; and (4) farming and food production.

## Zoonotic Diseases

Human well‐being is particularly vulnerable to zoonoses, animal diseases that can affect human populations. Richie Nimmo's (2010a, 2010b) work on the history of tuberculosis control and Frederick Keck's contribution to this special issue both highlight the importance of zoonotic diseases as organizing metaphors for understanding sociality. Zoonotic diseases trouble ideas of animality, humanity, and the “purity” of the social. While Nimmo describes how modern concepts of humanity were partly brought into being through the purification practices of zoonosis management and food controls in the late 19th century, Keck reveals how thinking about animal diseases influenced conceptualizations of the social among theorists, including Herbert Spencer, Emile Durkheim, and Claude Levi‐Strauss. Keck goes so far as to make the case for a parallel genealogy of animal health management and social anthropology.

It is not coincidental that medical anthropologists have begun exploring different forms of human animal health at a time when there is a growing concern with understanding life in the Anthropocene. Metaphors of entanglement, co‐production, and intersectionailty are becoming increasingly dominant in social theory as they are in epidemiology and other disciplines. Genese Sodikoff's contribution to this special issue highlights how different interpretations of the pathogenicity of rats gained traction during plague outbreaks in colonial and contemporary Madagascar. Asking how the deaths of rats act as signals of outbreaks, Sodikoff argues for a “veterinary semiotics,” in which understanding zoonotic events necessitates nonhuman participation. The point of veterinary semiotics is not to build more complex models but to construct more faithful “epidemic narratives,” attentive both to human suffering and to longue‐durée landscape change.

In contemporary popular discussions of zoonosis, including the recent outbreaks of Ebola or Lassa fever in West Africa, Avian influenza in Southeast Asia, and plague in Madagascar, metaphors such as spillover and containment put the threat that zoonotic diseases pose to humans into the dramatic language of security and catastrophe (see Garrett 1994; Quammen 2012). Zoonotic diseases are a powerful example of the shifts in biopolitics we described above, where increased awareness of interspecies vulnerability produce new kinds of governmental response (Porter 2013). Anthropologists working on zoonoses have critiqued the rationalities of preparedness that seek to predict and contain future disease outbreaks (Lakoff 2008; MacPhail 2014). Predictive, biosecurity‐oriented approaches to zoonosis can divert attention from the locally contingent “material proximities” that lead to outbreaks (Brown and Kelly 2015).

Alternative anthropological conceptions of zoonosis reflect a critique of the discourse of spillover and containment. If we are to escape the unilineal temporal logics that dominate in biosecurity frameworks, we must accept that species separation is not only ontologically impossible but undesirable. The sharing of blood, microbiota, food, and space is an essential part of becoming human—and becoming animal (Haraway 2008; Nading 2013, 2014a; Tsing 2015). Ethnographic approaches attuned to the multiple temporalities of multispecies relations are important for making sense of pathogenic entanglements and devising ways to live well with nonhuman others. Zoonotic events are not single moments in time or space; rather they reflect a recursive and ongoing set of interactions, mediated by the short‐term management of acute outbreaks, the longue‐durée of colonial and postcolonial biomedical practice, and the deep timescales of animal, insect, and microbial evolution.

## Veterinary Anthropology

Another place where those multiple temporal logics overlap is in veterinary medicine. Although veterinary knowledge is central in much of global health today, veterinary anthropology remains a remarkably underdeveloped area of medical anthropology. Recently, however, veterinary medicine has drawn ethnographic attention, pioneered in part by contributors to this collection (see Keck 2016). While the new interest in veterinary anthropology spans into animal studies and the history of science, it also raises questions pertinent to medical anthropology. Much of the work in this emerging area has reflected on the place of veterinary management in changing forms of global governance, particularly in epidemic situations. For example, Natalie Porter (2012) has examined the often contradictory role of veterinary specialists in the management of Vietnamese poultry flocks. Given geographical and historical differences across North and South Việt Nam, the shift of authority over bird health from family farmers to veterinarians in the context of avian influenza scares has been uneven, rather than fluid or instant (see Fortané and Keck 2015). Similar diversity in veterinary responsibility has been noted also in the bovine spongiform encephalaphy (BSE) and foot and mouth disease scares in the United Kingdom and Europe (Keck 2008; Law and Mol 2008).

But the scope of veterinary medicine extends well beyond new biopolitical formations. For example, what we know about human health has historically emerged in conversation with knowledge about the health of nonhuman animals (Keck, this special issue). The One Health approaches described above evolved partly from the ideas of scientists like Rudolf Virchow, who argued that “there is no scientific barrier, nor should there be, between veterinary medicine and human medicine” (Virchow 1872, cited in Saunders 2000: 203; Zinsstag et al. 2011). Even before there was a discrete field of veterinary medicine, as Keck (2016) has explained, physicians saw animal surgeries conducted in zoos as “analogous experiments” that offered insights into human health. The road to knowing and visualizing the diseased human body, then, has been historically routed through the animal body, and vice versa.

Indeed, medical anthropologists have as much to learn about care from ethnographic accounts of veterinary work with cats, dogs, and livestock as they do from accounts of human clinical encounters. Samantha Hurn and Alex Badman‐King's contribution to this collection suggests that the potential of veterinary anthropology for theory in medical anthropology is that veterinary ethics has less to do with what humans can or should do *to* animals, than what humans can or should do *for* and *with* animals. Questions of ethical accompaniment arise when we ethnographically explore how religious principles shape care for animals and their human companions, particularly at the end of life. Hurn and Badman King's work provides a useful counterpoint to One Health studies of veterinary practices, which have been focused on herd or flock management. For medical anthropologists, the breadth of veterinary anthropology—from the international institutional arrangements of One Health programs to the hyper‐local experience of living with domestic animals—places questions of intimate ethics into dialogue with the politics of risk and population health.

## Animals as Therapy, Animals in Therapy

Within medical anthropology, questions about care and ethics at the human‐animal interface are perhaps more familiar as a problem for biomedical research. Medical laboratory experiments depend on the participation of animals, from mice to nonhuman primates. Even so, many of us would not readily think of model organisms such as OncoMouse™ (Fujimura 1996; Haraway 1997); or the sentinel species that now aid in the control of West Nile, influenza, and other viral pathogens, as veterinary subjects (Lakoff and Keck 2013). When animals like sentinel chickens or mouse models are genetically modified or subjected to laboratory tests, they become parts of a clinical trial complex that sets the stage for the development of drugs and vaccines. Such animals are elements of what we might call a therapeutic assemblage.

As Carrie Friese and Joanna Latimer discuss in their contribution to this collection, questions of care and ethics shape how laboratory technicians become enrolled in the lives of experimental animals such as mice and rats. For the technicians with whom they work, care is first and foremost grueling physical labor. It is a repetitive, highly routinized endeavor, constrained by the physical makeup of cages and personal protective equipment as well as the familiar limitations of wage stagnation and fatigue. Yet these sometimes seemingly industrial routines are consistently disrupted by the particularities of interspecies interactions. To be effective caregivers, technicians must learn to detect the subtle behavioral cues of individual animals—even if, as “models,” these animals have been bred to meet rigid experimental standards.

The extent to which animals can become standardized therapeutic devices, of course, depends in part on the extent to which knowledge about animal bodies can be translated and rendered useful in human bodies. Laboratory animals straddle the lines between human and nonhuman, experimentation and production, potential therapy and potential harm. Animal labor and animal‐based resources are frequently obscured in the stories that we tell ourselves about human medicine (Svendsen 2017). In many ways, the integration of animals into therapeutic regimes and laboratory experiments reverses the logics of species separation and containment that characterize zoonotic disease control efforts such as One Health initiatives. The use of animal organs for human transplant (Sharp 2013) and the creation of experimental human–animal chimeras (Hinterberger 2016) open up new regulatory and moral dilemmas. Saving human lives involves not just the sacrifice of nonhuman animal models but also, it seems, the sacrifice of unitary ontological categories of human and animal.

Elsewhere, human–animal relationships themselves are increasingly being thought of as having therapeutic value. When relationships become therapeutic, species lines are arguably reinforced, rather than blurred. Studies have consistently shown not only that animals like cats and dogs provide companionship but also that such companionship can help reduce daily stress when people engage animals in walking, stroking, or agility exercises (McNicholas et al. 2005). In colleges and universities in the United States, therapy dogs have become campus fixtures. Student health services and libraries enlist therapy dogs to relieve students from the stresses of everything from sexual assault to homesickness to exam preparation. This kind of therapy straddles the line between psychology and customer service. The university's engagement of the animal as a provider of affective labor and the animal's propensity for “emotional intelligence” are impossible to disentangle. Echoing a theme raised in this collection by Hurn and Badman‐King's exploration of animal suffering and death, dogs and cats are now known also to play key roles in human end‐of‐life care, acting as companions for the dying and their families in hospice situations. Finally, dogs have been recognized for their ability to provide early warning for epileptic children vulnerable to seizures. These kinds of interspecies therapies join the familiar and longstanding use of service animals for the visually impaired to bring medical care into domestic interspecies economies previously dominated by agriculture, leisure, and hunting.

The therapeutic value of animal companionship comes in part from animals’ biological capacity to detect chemical changes in human bodies (as in the examples of end‐of‐life care and epilepsy) and in part from their role as beings that seem to have a knack for responding positively to human stress and anxiety. In mental and neurological health, companionship and interaction with animals has been identified as a breakthrough therapy for autistic children (Solomon 2010), and as providing a means for neglected children to mediate attachment to human adults (Carr and Rockett 2017). It is here that the human–animal relationship, rather than the presence of the animal alone, becomes most obviously therapeutic. For example, Roslyn Malcolm's recent work on “equine therapy” for autism shows how interaction with horses “opens up” children on the autism spectrum to forms of communication previously thought to be impossible or unlikely (Malcolm et al. 2017). Instead of communication or sociality being a capacity for well‐being that rests in the individual, Malcolm and others see it as essentially relational.

In human–animal health, of course, “animal therapy” is a two‐way street. In her contribution to this volume, Natalie Porter turns the above conversation on its head, asking how ideas of well‐being become integrated into dog training in animal rescue contexts. For Porter, the mind–body linkages taken for granted in human understandings of well‐being are challenged when emotional and bodily work are focused on dogs themselves. Her intervention vividly illustrates how thinking about health with animals challenges human‐centric anthropological perspectives.

Not all animal therapies require charismatic megafauna such as dogs, horses, and cats. The cultivation of relationships with less familiar (and seemingly less “friendly”) microorganisms is also being increasingly recognized as potentially therapeutic. Borne in part out of a recent explosion of interest in the human microbiome—the millions of viruses, bacteria, archaea, and fungi that populate human guts, skin, and hair—and partly out of a longstanding interest within alternative medicine in the therapeutic value of fermented, raw, rotten, or otherwise “living” foods, the notion that human bodies are themselves multispecies relationships is now nearly mainstream. When it comes to microbiota, therapy is a matter of reassembling or reconstituting those relationships, as Jamie Lorimer explains in his contribution to this collection. As Lorimer argues, do‐it‐yourself approaches to microbial health involve both formal and informal (and indeed sometimes illicit) exchange of worms, probiotics, and fecal matter. As in therapies involving megabiota, these exchanges require us to rethink the relationship between domestication and well‐being.

In one sense, purposely populating the gut with so‐called helpful microbes can be seen as an anthropocentric project. After all, the point of this particular form of domestication does not seem to develop the kind of affective or communicative bonds that characterize human relations with companion species. Much of the discourse about the microbiome, especially in the popular northern press, tends to presume that the subject that does the domestication is an individuated capitalist consumer (Nading 2016). As Lorimer (2016) and others have shown, however, engagement with human microbiota actually engenders quite a range of subject positions. After all, the key difference between enlisting microbes and worms as therapeutic helpmates and enlisting pharmaceutical drugs to do the same is that enlisting microbiota requires patients to relinquish the notion that they possess individual dominion over their bodies.

Microbiomic therapy is, in other words, an imprecise, iterative, and dynamic ecological undertaking, rather than an instrumental, mechanical one. It may come as no surprise, then, that medical anthropologists have been among the first to explicitly link the resistance of microbes to antibiotics with the resistance of insects and plants to pesticides (Orzech and Nichter 2008). Such ecological approaches to health upend neat scalar distinctions between the body, the community, and the globe (Nading 2013).

Even if an ontological separation between human and animal species is undermined within these kinds of therapeutic relationships, an epistemological separation between biomedical and nonbiomedical knowledge remains at stake. Often, these kinds of interspecies therapies are not prescribed or regulated by medical experts. Instead, they gain therapeutic traction through ad hoc experiments, word of mouth, journalistic reports, and the activism of patient and disability advocates. Animal therapy at both the macro‐ and microbiotic scales is a space where behavioral therapists, social care workers, activists, and volunteers push the definition of what it means to heal beyond the parameters of pharmaceutical and clinical practice. While medical anthropologists have often been critical of the unitary authority of biomedicine to explain health and illness, human–animal relations seem to exceed the biomedical gaze in quite different ways than, say, decisions about compliance with pharmaceutical prescriptions, or even decisions about whether to choose biomedical or alternative care. In other words, human relations with other animals can be therapeutic, but they are always more than that. We are always already entangled in such relations, and even in the case of microbes, therapeutic value is only part of the story of domestication and only one aspect of the way in which we become human through our lived relations with other animals.

## Humans, Animals, Food

Nowhere is this more obvious than in the fourth and final dimension of human animal health, that of food and nutrition. For medical anthropologists, the question of food and health raises some immediate and well‐known concerns about the entanglement of pathogens, humans, and other species. Animal suffering in industrial farms has been seen mostly as an ethical issue rather than a medical or sociological one (Grandin 2015; Pachirat 2011), but the implications of meat production for human health have recently become more direct concerns for social scientists. At a basic level, exposure to occupational risks in high‐intensity agricultural production in the United States is unevenly distributed along lines of class, race, and nationality (Holmes 2013; Horton 2016). At a slightly more complex level, the problem of antibiotic resistant bacteria has been linked to the overuse of antibiotics in industrial meat systems (Orzech and Nichter 2008). In food systems, then, human laboring populations, animal populations, and microbial populations are all different kinds of “lively capital” (Haraway 2008). Their reproductive lives are over‐determined by the push for intensified accumulation.

Beyond the industrial systems of the global North, animal death has also been a point of departure for human animal health. The culling of livestock and poultry has become a reliable element of global health interventions, evident during the European BSE and foot and mouth scares and the SARS and “swine” influenza outbreaks (Law 2010). Racist and otherwise misleading information about the dangers of animal, bird, and rodent protein (or “bushmeat”) consumption severely impeded public buy‐in to the global effort to stop the West African Ebola epidemic that began in 2014 (Bonwitt et al. 2018; McGovern 2014; Richards 2016, 80–81). The bushmeat ban was criticized for diverting attention from other more important public health efforts (Wilkinson and Leach 2015) and for leaving people without other options for securing food in contexts where wild animal protein forms a significant part of people's diets (van Vliet and Mbazza 2011).

In his contribution to this collection, Alex Blanchette uses the problem of microbially impregnated dust emanating from industrial hog farms in the U.S. Midwest to explore how the registers of the political economic, the symbolic, and the biological might be joined together. The production of this unique form of living industrial waste provides insights into the ways in which banal and routinized (if also incredibly dangerous) labor takes on what Blanchette calls “planetary significance.” Alongside his informants, Blanchette contemplates the necessity of becoming “anesthetized” to the presence of potentially pathogenic dust. The fecal dust that envelops the hog operations contains a dry, crunchy, smelly history of interspecies labor relations, as well as warnings about a future of unchecked antimicrobial resistance (Choy and Zee 2015). Vulnerability here is less a matter of risk that must be calculated and managed by experts than a matter of concern that must be articulated and debated by workers, residents, and social scientists seeking to come to terms with the embodied and environmental consequences of late industrialism (Fortun 2012).

Blanchette's contribution raises further questions for medical anthropology. Even though an interest in the racialized and gendered burden of occupational health risk in the food system has already made its way into many discussions within our subdiscipline, what would happen, ethnographically, ethically, and otherwise, if we started to think about the *work* that bees, pigs, cattle, and sheep do? What would it mean to treat such animals not only as patients but also as laborers? A multispecies medical anthropology that focuses not only on the positive valences of human–animal relations but also on its more problematic and even antagonistic aspects can contribute to expanding the scope occupational health as an organizing discourse. What forms of interspecies solidarity and conflict, we might ask, emerge between human and animal workers on farms and killing floors (see Blanchette Forthcoming)?

Of course food systems are more than farms. As Michelle Murphy (2013) has shown, the reproductive and cognitive effects of exposure to toxic industrial chemicals such as PCBs are particularly acute in fisheries. Toxics have multispecies epigenetic consequences. Their effects take hold across multiple generations—of fish and humans. This observation seems important to our discussion, since it reminds us that animals are many things. They are sometimes companions, sometimes sentinels, sometimes food, sometimes workers, and sometimes some combination of all these things.

## Conclusion

To provisionally map out some of the sites identified in the contributions to this special issue, Table One provides a simplified visualization of significant relationships between theoretical and empirical dimensions of human animal health.

**Table:** 

EMPIRICAL					
		ZOONOTIC DISEASES	VETERINARY ANTHROPOLOGY	ANIMAL THERAPEUTICS	FARMING AND FOOD
THEORECTICAL	BIOPOLITICS	Risk and preparedness Purifying the “social” Outbreak management	Health of the herd ‘One Health’ interventions Animals as sentinels	Animals as caregivers in welfare regimes	Regulation of animal lives and deaths Welfare of farmed animals
	CARE	Livestock/poultry in/as kin relations Animal protection (e.g., anti‐culling protests)	Care for animals Euthanasia	Veterinary medicine Therapy animals	Ethical treatment Local, organic, free range movements
	ECOLOGY	Emerging diseases	Management of rabies, ticks, and parasites, including zoonoses like Toxoplasmosis	Transgenic/GM animals	Farmed landscapes Animal waste
	POLITICAL ECONOMY	One World One Health^TM^	Veterinary marketing, pharmaceuticals	Markets for animal‐based therapeutic products	Farming, including intensive and small scale

This table presents a somewhat capacious outline for future inquiry. We present this collection as an invitation to engage and debate an emerging approach to human animal health. Taken together, the contributions remind us that human animal relations are anything but incidental to medical anthropology. They are a central component not only of its history but also of its leading edge. What is particularly invigorating about the conversation that follows is its inclusion of both scholars familiar with medical anthropology and scholars for whom medical anthropology has opened new avenues of thinking. In the opening pages of this introduction, we suggested that the discomfort of these “newcomers” with the discourse and practice of medical anthropology was, in the end, a productive one. We invite readers to open themselves to that productive discomfort, to rethink ecology, biopolitcs, and care, and to join us in reimagining health and well‐being as more than human concerns.
